# Cell walls as a stage for intercellular communication regulating shoot meristem development

**DOI:** 10.3389/fpls.2015.00324

**Published:** 2015-05-11

**Authors:** Toshiaki Tameshige, Yuki Hirakawa, Keiko U. Torii, Naoyuki Uchida

**Affiliations:** ^1^Institute of Transformative Bio-Molecules (WPI-ITbM), Nagoya University, Nagoya, Japan; ^2^Department of Biology, University of Washington, Seattle, WA, USA; ^3^Howard Hughes Medical Institute, University of Washington, Seattle, WA, USA

**Keywords:** auxin, cell wall, cytokinin, intercellular communication, organ primordia, peptide hormone, physical stress, shoot apical meristem

## Abstract

Aboveground organs of plants are ultimately derived/generated from the shoot apical meristem (SAM), which is a proliferative tissue located at the apex of the stem. The SAM contains a population of stem cells that provide new cells for organ/tissue formation. The SAM is composed of distinct cell layers and zones with different properties. Primordia of lateral organs develop at the periphery of the SAM. The shoot apex is a dynamic and complex tissue, and as such intercellular communications among cells, layers and zones play significant roles in the coordination of cell proliferation, growth and differentiation to achieve elaborate morphogenesis. Recent findings have highlighted the importance of a number of signaling molecules acting in the cell wall space for the intercellular communication, including classic phytohormones and secretory peptides. Moreover, accumulating evidence has revealed that cell wall properties and their modifying enzymes modulate hormone actions. In this review, we outline how behaviors of signaling molecules and changes of cell wall properties are integrated for the shoot meristem regulation.

## Introduction

Plants elongate stems upward, expand leaves widely to perceive the sunlight, and form flowers for reproduction. These aboveground organs are all developed from the shoot apical meristem (SAM), the proliferative tissue located at the top of the stem. The SAM contains a population of stem cells, which are characterized by two functions: providing new cells for organ/tissue formation and maintaining their own undifferentiated population. In typical eudicots, the SAM is composed of three clonally distinct cell layers, L1, L2, and L3, from outermost to inner cell layers (Steeves and Sussex, 1989; Bowman and Eshed, 2000). Cells in the L1 and L2 layers divide anticlinally, thus serving as the source of epidermal/subepidermal tissues that cover whole aerial parts. On the other hand, cells in the L3 layer divide in various planes to give rise to other inner tissues. The SAM can also be divided into three zones with different anatomical properties: the central zone (CZ) at the top, the peripheral zone (PZ), and the rib zone (RZ) beneath the CZ (Steeves and Sussex, 1989; Bowman and Eshed, 2000). The CZ contains less mitotically active stem cells, replenishing cells for the surrounding PZ and RZ, where cells divide more rapidly and give rise to lateral organs and stems. Primordia of leaves and flowers are formed successively at the periphery of the SAM with a constant divergent angle, resulting in beautifully arranged patterns of the lateral organs (phyllotaxis; Jean, 1994). During organ initiation, cells in a corresponding part of the PZ swell and divide to form a bulge-shaped primordium, and they further proliferate to form a lateral organ.

Intercellular communication plays crucial roles for the coordination among cells and tissues in the shoot apex to achieve elaborate morphogenesis of the plant body. Classically, a series of surgical experiments have predicted the presence of such signals (Steeves and Sussex, 1989). Recent molecular studies have revealed that not only small molecular weight phytohormones but also secretory peptides mediate intercellular signaling. Furthermore, accumulating evidence has highlighted the importance of the extracellular cell wall space for hormone actions. In this review, we outline intercellular communication in the shoot apex, especially focusing on events in the extracellular space, such as hormone transport, metabolism and the dynamics of the cell wall properties.

## Auxin Transport through the Extracellular Space is Regulated by Cell Wall Properties

Among phytohormones, auxin has been the best characterized in shoot development (Davies, 1995). Auxin regulates a range of developmental processes in specific cells. The following two perception systems for auxin have been reported to mediate the auxin action. Firstly, a group of nuclear-localized receptors encoded by *TIR1* (*TRANSPORT INHIBITOR RESPONSE 1*)/*AFB* (*AUXIN F-BOX*) genes perceive auxin. In the presence of auxin, the receptors activate ARF (AUXIN RESPONSE FACTOR) family transcription factors to regulate their target genes (Guilfoyle and Hagen, 2007; Salehin et al., 2015). Secondly, an extracellular receptor, ABP1 (AUXIN BINDING PROTEIN1), binds to auxin (Löbler and Klämbt, 1985). Auxin activates ROP (RHO OF PLANTS) signaling in an ABP1-dependent manner, which in turn regulates clathrin-coated vesicle trafficking and cytoskeleton dynamics (Robert et al., 2010; Xu et al., 2010; Nagawa et al., 2012; Grones and Friml, 2015). In the L1 layer of the SAM, auxin accumulation induces formation of lateral organ primordia. Thus the auxin distribution pattern determines the phyllotaxis. The accumulated auxin in the L1 layer is subsequently transported into underlying L2 and L3 tissues (Benková et al., 2003; Reinhardt et al., 2003; Heisler et al., 2005; Bayer et al., 2009). This auxin flow induces vascular differentiation that eventually connects a vascular network from the primordium to stem vasculatures (Bayer et al., 2009). In a growing primordium, auxin is again accumulated in a dotted pattern in the marginal epidermis, inducing formation of leaflets, lobes, serration and venation (Hay et al., 2006; Scarpella et al., 2006; Barkoulas et al., 2008).

The major natural auxin, indole-3-acetic acid (IAA), is synthesized by intracellular enzymes, which are expressed with specific spatio-temporal patterns (Zhao, 2008; Chandler, 2009). Then, intercellular transport of auxin from the biosynthesis sites determines its distribution pattern. Auxin transport is facilitated by plasma membrane-localized auxin efflux carrier protein, PIN1 (PIN-FORMED 1). Genetic and chemical perturbation of PIN1 impairs the local auxin accumulation and following developmental events: lateral organ initiation (Okada et al., 1991; Reinhardt et al., 2000), phyllotaxis (Guenot et al., 2012), vascular patterning (Scarpella et al., 2006), and leaf margin morphogenesis (Hay et al., 2006). In these events, PIN1 proteins localize at a specific part of the plasma membrane and the coherent PIN1 polarization in a group of cells causes directional auxin flow toward the point of its accumulation (Boutté et al., 2007; Nakamura et al., 2012; Luschnig and Vert, 2014). Studies on mechanisms for the PIN1 polarization proposed that PIN1 proteins preferentially localize toward the most auxin-rich neighboring cell or toward the cell wall where the auxin flux is the highest (Jönsson et al., 2006; Smith et al., 2006; Stoma et al., 2008; Bayer et al., 2009). It was also proposed that PIN1 proteins concentrate toward the cell wall that exhibits the highest stress (Heisler et al., 2010; Nakayama et al., 2012; Braybrook and Peaucelle, 2013). These two types of models have started to be integrated by recent studies and the details of this integration will be described in the later section of this review on physical properties of the cell wall. These findings highlight a novel role for cell walls in the regulation of auxin distribution. It has been discussed that, in the SAM and leaf primordia, PIN1 dynamics might be differently regulated between the epidermal L1 layer and the inner L2/L3 tissues (Tsugeki et al., 2009; O’Connor et al., 2014). Because the proposed models for stress responses only dealt with phenomenon in the L1 layer, it would be important to investigate whether these models can be applied to the L2/L3 tissues.

The auxin distribution pattern also depends on its influx into cells since mutations in auxin influx carrier genes lead to abnormal phyllotaxis (Cheng et al., 2007; Bainbridge et al., 2008). Interestingly, the auxin influx rate is affected by a chemical property of cell wall space. Major auxin influx carriers, AUX1/LAX (LIKE-AUX1) proteins, catalyze H^+^/IAA-symport (Yang et al., 2006; Boutté et al., 2007; Vieten et al., 2007). Therefore, this process largely depends on the pH difference across the plasma membrane. In addition, the auxin influx occurs by diffusion. Because the extracellular pH is acidic (∼5.5) and the cytosolic pH is neutral (∼7.0), a substantial amount of IAA is protonated in the extracellular space and is ionized in the cytosol. As only the protonated form is freely transported through lipophilic plasma membranes because of its neutral charge, the diffusion is unidirectional from outside of the cell to the inside. Plants indeed alter the extracellular pH to create the auxin gradient in hypocotyls by utilizing plasma membrane-localized H^+^-ATPases (Hohm et al., 2014).

## Cytokinin also Acts for Intercellular Communication

Cytokinins (CKs) are adenine derivatives, which also act as a phytohormone playing significant roles in the SAM activity and organ morphogenesis. Indeed, two key transcription factors for the SAM regulation, SHOOT MERISTEMLESS (STM) and WUSCHEL (WUS), control the CK homeostasis and signaling, highlighting the importance of CK for the regulation (Yanai et al., 2005; Gordon et al., 2009). CK also impacts phyllotaxis (Giulini et al., 2004), lateral organ initiation (Yoshida et al., 2011), leaf margin morphogenesis (Greenboim-Wainberg et al., 2005; Shani et al., 2010; Efroni et al., 2013), and leaf vein patterning (Werner et al., 2003).

Cytokinins is transported not only locally among neighboring cells but also distantly between shoot and root. CK moves from shoot to root symplastically via phloem and from root to shoot apoplastically via xylem (Kudo et al., 2010). The intercellular CK movement is facilitated by both transporters and passive diffusion. ENT (EQUILIBRATIVE NUCLEOSIDE TRANSPORTER) and PUP (PURINE PERMEASE) proteins act as importers (Bürkle et al., 2003; Li et al., 2003; Hirose et al., 2005; Sun et al., 2005; Cedzich et al., 2008; Qi and Xiong, 2013). On the other hand, ABCG14 (ATP-BINDING CASSETTE TRANSPORTER SUBFAMILY G14) was implicated as an exporter candidate. Some of ENTs and PUPs are expressed in shoot tissues and their mutations change shoot morphologies. However, a large and complex redundancy among these family members hampers comprehensive analysis to further investigate roles for these transporters. The *ABCG14* loss-of-function mutation alters the SAM size and growth rate. Interestingly, grafting experiments indicate that the *ABCG14* expression in root is essential for shoot development (Ko et al., 2014), demonstrating the significance of the CK translocation between shoot and root for the shoot development. On the other hand, the importance of the CK transporters for the local movement is still unclear. At least, in contrast to auxin transport, highly directional transport is not assumed and simple diffusion of CK is predicted owing to its high membrane permeability (Laloue et al., 1981; Kudoyarova et al., 2014). Accordingly, recent mathematical models have reconstituted expression patterns of CK-responsive genes for the SAM regulation and vascular formation by assuming only simple and constant diffusion for the CK transport (Chickarmane et al., 2012; De Rybel et al., 2014).

Enzymes responsible for CK biosynthesis are localized in cytosol and intracellular organelles (Kieber and Schaller, 2014), while the degradation is catalyzed by CKX (CYTOKININ DEHYDROGENASE) family members, some of which are reported to act as extracellular proteins (Motyka et al., 2003; Werner et al., 2003; Kieber and Schaller, 2014). In addition, CKX members show different substrate preferences and expression patterns (Werner et al., 2003; Gajdošová et al., 2011; Köllmer et al., 2014). These imply that each CKX degrades a specific pool of CK according to their particular spatial distribution and substrate preference. While it was shown that the mutant of a putative extracellular CKX, CKX5, displays defects of the SAM and stem morphologies (Bartrina et al., 2011), the biological significance of the extracellular CK degradation is still largely obscure. It was reported that, in moss, the extracellular amount of CK correlates with some developmental alterations rather than its intracellular amount (von Schwartzenberg et al., 2007).

## Crosstalks between Auxin- and CK-Related Regulations of SAM Development

As mentioned above, auxin and CK play important roles in the shoot apex development. Interestingly, in recent years, crosstalk between these hormone pathways has been revealed (Su et al., 2011; Murray et al., 2012; Schaller et al., 2015). For example, PIN1-dependent auxin accumulation leads to the down-regulation of *STM* that promotes CK biosynthesis (Furutani et al., 2004; Heisler et al., 2005; Yanai et al., 2005). Auxin also modulates the cellular responses to CK by changing the expression of negative regulators of CK signaling cascade (Zhao et al., 2010; Besnard et al., 2014); up-regulating *ARR7* (*ARABIDOPSIS RESPONSE REGULATOR 7*) and *ARR15* and down-regulating *AHP6* (*ARABIDOPSIS HISTIDINE PHOSPHOTRANSFER PROTEIN 6*). On the other hand, CK affects the *PIN1* expression level in the SAM, suggesting that CK may modulate the auxin transport rate (Lee et al., 2009). In addition, the PIN1 polarization is influenced by the CK in the roots (Bishopp et al., 2011; Marhavý et al., 2014), though it remains to be examined whether the same mechanism also functions in the SAM. Mutual regulation between auxin and CK provides robustness and dynamics of the SAM homeostasis (Besnard et al., 2014). Meanwhile, these complex systems make it difficult to understand the whole picture only by traditional types of experiments and observations. Computer-based mathematical modeling approaches may provide solutions to this issue. Indeed, a successful case has been reported on the mutual regulation between auxin and CK in embryogenesis (De Rybel et al., 2014).

## Peptide Hormones and Receptors Regulating SAM Development

Peptide hormones are another important class of signaling molecules mediating intercellular communication through the cell wall space. Since the discovery of 18 amino-acid systemin as a defense response signal in tomato (Pearce et al., 1991), a variety of signaling peptides have been identified in plants. All of these peptides are produced via proteolytic cleavage of precursor proteins encoded by specific genes. Although the systemin was shown to localize in the cytosol (Narváez-Vásquez and Ryan, 2004), most of the others are presumed to be secreted into the apoplasts by the N-terminal signal peptide (Matsubayashi, 2014). These secreted peptides bind to specific cell-surface-localized receptors at target cells and affect cellular behaviors such as cell division, cell morphogenesis and cell fate determination. Peptide hormone genes are classified into distinct families based on the similarity in their mature peptide sequences. Each family contains multiple genes, which produce structurally similar or even identical mature peptides. Post-translational modification is also critical for their bioactivities. Secreted peptides often contain proline, tyrosine and/or cysteine residues post-translationally modified by specific enzymes (Komori et al., 2009; Ogawa-Ohnishi et al., 2013; Matsubayashi, 2014). Also, some proteases are responsible for the proteolytic cleavage in the maturation steps (Berger and Altmann, 2000; Srivastava et al., 2008; Tamaki et al., 2013; Engineer et al., 2014).

In the SAM, CLAVATA3 (CLV3) peptide, a member of CLE (CLV3/ESR-related) peptide family, mediates intercellular communications maintaining stem cell homeostasis (Cock and McCormick, 2001). The structure of the mature CLV3 peptide was determined as a 13 amino-acid peptide containing two hydroxyproline residues, one of which was further triarabinosylated (Ohyama et al., 2009). The CLV3 peptide is produced by stem cells in the CZ and perceived by its receptors at neighboring cells (Fletcher et al., 1999; Brand et al., 2000; Schoof et al., 2000). Leucine-rich repeat (LRR)-type receptors participate in the CLV3 signaling pathway. CLV1 is an LRR receptor kinase (LRR-RK), which binds to CLV3 (Ogawa et al., 2008; Ohyama et al., 2009). The BAM (BARELY ANY MEISTEM) proteins are close relatives of CLV1. Genetic and biochemical studies implied that BAMs also possess the binding capability to CLV3 peptides (DeYoung et al., 2006; DeYoung and Clark, 2008; Shinohara et al., 2012). In addition, other membrane-localized proteins including CLV2, CRN (CORYNE)/SOL2 (SUPPRESSOR OF LLP1 2) and RPK2 (RECEPTOR-LIKE PROTEIN KINASE 2) are involved in the CLV3 pathway (Miwa et al., 2008; Müller et al., 2008; Kinoshita et al., 2010). These proteins form various combinations of receptor complexes to mediate CLV3 signaling (Bleckmann et al., 2010; Guo et al., 2010; Kinoshita et al., 2010; Zhu et al., 2010). The CLV3 signal represses the expression of *WUS* that encodes a transcription factor promoting the proliferation of CLV3-producing stem cells (Mayer et al., 1998; Brand et al., 2000; Schoof et al., 2000). This local feedback is integrated with CK and auxin signaling, thus maintaining the dynamic SAM architecture (Leibfried et al., 2005; Gordon et al., 2009; Zhao et al., 2010; Chickarmane et al., 2012).

Phytosulfokine (PSK) is a disulfated five amino-acid peptide identified first in the Asparagus cell culture as a factor that promotes cell proliferation (Matsubayashi and Sakagami, 1996). Five genes produce the identical PSK peptides in Arabidopsis and are expressed in almost all tissues (Matsubayashi et al., 2006). PLANT PEPTIDE CONTAINING SULFATED TYROSINE 1 (PSY1), 18 amino acid glycopeptide, displays a biological activity similar to PSK (Amano et al., 2007). A PSK receptor (PSKR) was biochemically identified from carrot *Daucus carota* microsomal fractions (Matsubayashi et al., 2002). Arabidopsis homologs of DcPSKR act as receptors for PSK or PSY1, being expressed in a broad range of tissues including the SAM (Matsubayashi et al., 2006; Amano et al., 2007). Genetic studies of the receptors highlight the importance of PSK/PSY1 signaling in the cell proliferation, expansion and wound repair (Matsubayashi et al., 2006; Amano et al., 2007).

ERECTA (ER)-family LRR-RKs regulate various aspects of growth and development such as stem growth, stomatal patterning and the SAM regulation (Torii et al., 1996; Shpak et al., 2004; Uchida et al., 2011, 2013; Chen et al., 2013). The loss of the entire family activities impairs the SAM homeostasis, leading to an increase in stem cell population (Uchida et al., 2013). Because the SAM of *er*-family mutant shows enhanced CK response and abnormal auxin accumulation pattern (Chen et al., 2013; Uchida et al., 2013), ER-family signaling is thought to have crosstalks with CK and auxin pathways. Although a ligand for the ER family in the SAM regulation is still unknown, the ligands for stem growth and stomatal development have been identified: All of them belong to the same family of peptide hormones, the EPF (EPIDERMAL PATTERNING FACTOR)/EPFL (EPF-LIKE) family (Hara et al., 2007, 2009; Hunt and Gray, 2009; Abrash and Bergmann, 2010; Hunt et al., 2010; Kondo et al., 2010; Sugano et al., 2010; Abrash et al., 2011; Uchida et al., 2012, 2013). The EPF/EPFL family encodes cysteine-rich secretory peptides, which possess intramolecular disulfide bonds essential for the conformation of their bioactive mature forms (Kondo et al., 2010; Sugano et al., 2010; Ohki et al., 2011). A yet unknown ligand for ER family for the SAM regulation may also be a member of this family.

To date, more than 15 peptide hormone families have been described (Kondo et al., 2014). Though each family consists of multiple paralogs, roles for most of them are still obscure. Also, it is expected that yet unidentified peptide hormone genes can be found in genomes. It would be an important challenge to identify further secretory peptides, which participate in the shoot apex regulation.

## Cell Wall Loosening Proteins as Key Factors in Organ Formation

Plant cells need to extend their cell walls during growth and development. Cell wall extensibility is enhanced by growth promoting phytohormones, including auxin and CK (Cleland, 1958; Thomas et al., 1981), and has started to be recognized as a key factor that determines developmental patterns and cell fates. The first breakthrough came from experiments using an apoplastic protein, EXP (EXPANSIN; Fleming et al., 1997; Pien et al., 2001), which relaxes cellulose microfibril network by binding to xyloglucan (Wang et al., 2013). The EXP expression increases in newly formed leaf primordia. Because its wall-loosening activity exhibits an acidic pH optimum, it is suggested that auxin accumulation in leaf primordia further increases the EXP activity (McQueen-Mason and Cosgrove, 1994; Cleland, 2001). It was also reported that local application of exogenous EXP proteins as well as artificial expression of *EXP* induces leaf formation on the SAM (Fleming et al., 1997). These indicate that local loosening of the cell wall triggers organ initiation. Auxin is also known to promote demethylesterification of pectin (Bryan and Newcomb, 1954; Braybrook and Peaucelle, 2013), and another secreted enzyme, PME (PECTIN METHYLESTERASE), was shown to loosen cell walls by demethylesterifying pectin (Peaucelle et al., 2008, 2011a). PME induces the organ initiation when applied to the SAM surface as well as EXP (Peaucelle et al., 2008, 2011a). Furthermore, a member of PGX (POLYGALACTURONASE) family regulates the organ initiation pattern by cleaving pectins (Xiao et al., 2014). These findings highlight the significance of the dynamic nature of cell wall extensibility during organ development.

The importance of the cell wall loosening has also been characterized in the stem cell regulation in moss. During stem cell induction triggered by leaf excision in Physcomitrella, some cell wall loosening enzymes are up-regulated (Sakakibara et al., 2014). Meanwhile, transient expression of *EXP* enhances the stem cell formation (Sakakibara et al., 2014). While the underlying molecular mechanisms may not be conserved between the SAM of vascular plants and stem cells of mosses, these observations emphasize the importance of cell wall extensibility in cell fate determination.

## Cell Wall Loosening Changes Microtubule Orientation and Auxin Pattern in the SAM

**FIGURE 1:**
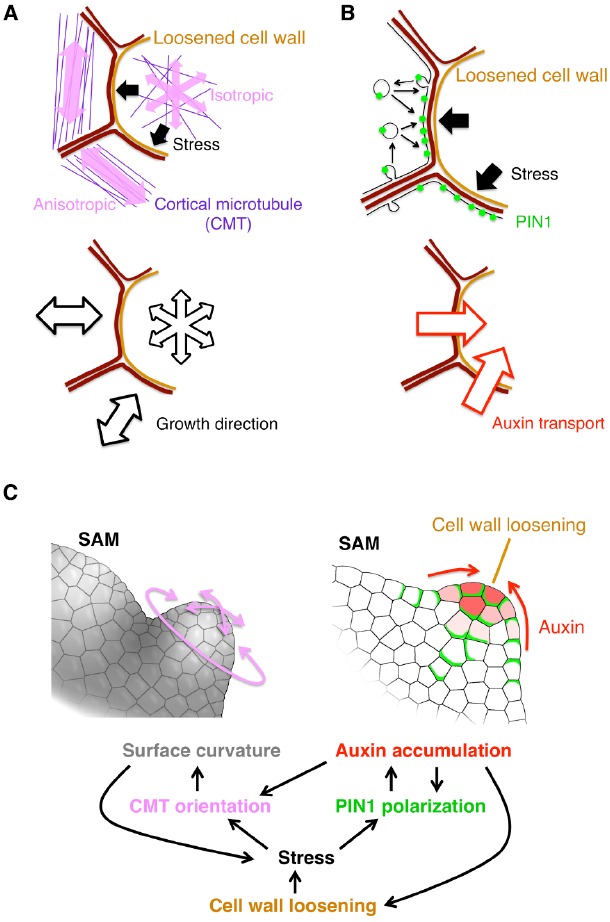
**Current model for organ initiation and the role for cell wall loosening. (A,B)** The stress from the cell with loosened cell wall leads to reorientation of cortical microtubule (CMT) **(A)** and PIN1 polarization toward the loosened wall **(B)** in neighboring cells. **(C)** Regulatory network among cell wall loosening, CMT and PIN1. Details are mentioned in the text.

Cell wall extensibility is a physical property. Therefore, it has been a key question how such a physical property is decoded as a developmental signal to change cellular behaviors. The physical force generated by cell wall loosening could provide an answer. In a growing tissue, cells are constrained by the cell wall. Therefore, cell wall loosening results in cell swelling and pushing the neighboring cells. Recent studies revealed that such pushing force regulates cellular behaviors by affecting the following two factors: cortical microtubule (CMT) orientation and PIN1 protein localization (Figure 1).

The first player, CMT, is a type of cytoskeleton microtubules located under the plasma membrane. In general, the CMT orientation correlates with the stress direction in various plant tissues (Green and King, 1966; Williamson, 1990; Cleary and Hardham, 1993; Wymer et al., 1996; Fischer and Schopfer, 1998). By combining live imaging of CMT, computation of stress properties and laser manipulation techniques, it was shown that, in the SAM, the stress direction determines the CMT orientation (Hamant et al., 2008; Figure 1A). It was also revealed that KATANIN proteins catalyze depolymerization of crossing microtubules to form parallel CMT arrays (Uyttewaal et al., 2012). Since the CMT orientation directs the movement of cellulose synthase complex, cellulose microfibril orients in parallel to the CMT (Paredez et al., 2006). This cellulose microfibril array then restricts the cell expansion to its perpendicular direction (Figure 1A). Thus, cell wall loosening in one cell confers the growth anisotropy on its surrounding cells in response to the initial stress direction, leading to a local outgrowth of the tissue. Moreover, the uneven surface curvature formed by the local outgrowth, and, in turn, renders an anisotropic stress from the inner tissue to the outer tissue, forming a positive feedback circuit that contributes to continuous tissue outgrowth (Hamant et al., 2008; Figure 1C, left).

The second player is the auxin efflux carrier PIN1 as described before in the earlier section. Wall loosening in a cell can cause local stress in the neighboring cells, and it has been proposed that the PIN1 protein is preferentially localized to a part of plasma membrane where the contacting cell wall is the most stressed (Heisler et al., 2010; Figure 1B). A plausible model for this characteristic localization is as follows. PIN1 distribution is dynamically regulated through exocytosis and endocytosis, which increase and decrease the local PIN1 amount in the plasma membrane, respectively. Exocytosis is activated near the highly stressed portion of cell walls where the tension of plasma membrane is also high, thus leading to PIN1 enrichment there (Nakayama et al., 2012; Figure 1B). The resulting PIN1 localization facilitates transport of auxin into the wall-loosening cell (Figures 1B,C, right). Auxin, in turn, stimulates PME and EXP activities to further drive the cell wall loosening (Cleland, 2001; Braybrook and Peaucelle, 2013; Figure 1C, right). Thus, PIN1 mediates this positive feedback to enhance both cell wall loosening and local auxin accumulation (Braybrook and Peaucelle, 2013; Figures 1B,C, right).

Taken together, cell wall loosening triggers organ formation by the following two feedback mechanism (Sampathkumar et al., 2014). One is the feedback between the CMT anisotropy and the SAM geometry (Figures 1A,C left). The other is between PIN1-dependent auxin transport and auxin-induced wall loosening (Figures 1B,C right). Because the auxin accumulation increases the CMT isotropy (Sassi et al., 2014), these two feedback circuits are interconnected (Figure 1C). Therefore, any of auxin accumulation, cell wall loosening and CMT anisotropy change can initiate organ primordium formation. It would be interesting to investigate which of them is the earliest event in nature.

In addition, it was recently reported that a membrane-bound kinase, D6PK (D6 PROTEIN KINASE), phosphorylates PIN1 to control its localization and, vice versa, auxin promotes the polar distribution of D6PK at the plasma membrane, suggesting another feedback mechanism for the auxin-PIN1 mutual regulation (Zourelidou et al., 2009; Willige et al., 2013; Barbosa et al., 2014; Figure 1C). It would be interesting to investigate whether and/or how this D6PK-dependent mechanism and the above-mentioned stress-mediated mechanisms are integrated.

## Remaining Questions on Relationships between Cell Wall Loosening and Organ Development

Whereas roles for cell wall loosening proteins in organ formation are evident and the underlying mechanisms have begun to unveil, questions and problems to be challenged still remain. Firstly, methods to directly measure physical stress in the SAM and initiating organs need to be further developed, as our current knowledge on the stress has been all indirectly estimated from measurements of other physical properties: the tissue geometry by scanning electron microscopy (Hamant et al., 2008), the elasticity by atomic force microscopy (Peaucelle et al., 2011a) and the cellular volume expansion caused by manipulation of osmotic pressure (Kierzkowski et al., 2012). Therefore, direct measurements of the stress are ultimately required for comprehensive understanding. Developing new applications of micro-manipulation tools (e.g., microfluidic device, optical tweezers and magnetic nanoparticles) might help to overcome this issue.

Secondly, little is known about mechanisms by which cells directly perceive the physical stress in the SAM. It has been reported that, in other parts such as roots and pollen tubes, loss of function of some receptor-like proteins (THESEUS1, FERONIA, ANXUR1/2, and Receptor-like protein 44) and putative mechanosensitive channels (MCA1/2) alters cellular responses to physical stress or changes in cell wall polysaccharide composition (Hématy et al., 2007; Nakagawa et al., 2007; Boisson-Dernier et al., 2013; Shih et al., 2014; Wolf et al., 2014). Though these proteins could be candidates for sensors to monitor physical stress or cell wall conditions (Cheung and Wu, 2011; Höfte, 2015), direct evidence is still lacking. It also remains to examine whether these proteins contribute to the shoot apex regulation.

Thirdly, though the *pin1* mutation and the treatment of auxin transport inhibitors impair leaf morphogenesis and phyllotaxis (Kawamura et al., 2010; Guenot et al., 2012), they do not block the leaf initiation itself. Therefore, the PIN1-independent organ initiation machinery must exist. Some studies implicated that this machinery is driven largely by local auxin biosynthesis by YUCCA proteins (Cheng et al., 2007), auxin signal transduction by ARF5 (Schuetz et al., 2008), and dynamics of CMT array regulated by auxin through ABP1 and KATANIN (Sassi et al., 2014). These suggest that there should be a PIN1-independent but auxin-dependent process for the organ initiation. Activities of auxin influx carriers, AUX1/LAX, might be able to organize auxin distribution to some extent even without PIN1 proteins. Alternatively, as discussed above, any change in cell wall integrity, SAM surface geometry or physical stress might trigger the initiation of organ formation. Further analysis of the *pin1* mutant is required to understand these processes.

Lastly, compared with the SAM regulation, there is only limited information as to whether and/or how cell wall loosening is regulated in growth and morphogenesis of other tissues. Some studies have shown that *EXP* induces the leaf lobe formation and increases the total leaf size (Pien et al., 2001; Sloan et al., 2009; Goh et al., 2012) and also that *PME* promotes stem elongation (Peaucelle et al., 2011b). However, the involvement of auxin and CMT is unclear in these processes. To further understand the developmental roles for the cell wall loosening, it is important to characterize similarities and differences of molecular mechanisms in different developmental events.

## Concluding Remarks

In this review, we outlined current knowledge on the regulation of shoot apex development, especially highlighting signaling molecules, physicochemical properties of cell walls and their modifiers in the extracellular space. A great variety of their actions and interactions demonstrate the importance of the cell wall space as a field for intercellular communication more than just a wall that separates individual cells. This viewpoint is indispensable to understand how plants have evolved their own multicellular body whose cells are interconnected by cell walls in sharp contrast to the animal body. Further technical developments in imaging, measurement, manipulation and mathematical modeling will provide more insights into the dynamics and functions of the cell wall space.

### Conflict of Interest Statement

The authors declare that the research was conducted in the absence of any commercial or financial relationships that could be construed as a potential conflict of interest.
